# Lipoic Acid Enhances the Defense Capability of Citrus Fruits to Blue Mold Caused by *Penicillium italicum*

**DOI:** 10.3390/foods14060987

**Published:** 2025-03-14

**Authors:** Zhihong Lu, Min Hong, Rikui Wang, Yu Feng, Shiming Cheng, Mingyang He

**Affiliations:** 1 Citrus Research Institute, Southwest University/Chinese Academy of Agricultural Sciences, Chongqing 400712, Chinaxndxgyshm@swu.edu.cn (M.H.); wangrikui@cric.cn (R.W.);; 2 Key Laboratory of Quality and Safety Control of Citrus Fruits, Ministry of Agriculture and Rural Affairs, Southwest University, Chongqing 400712, China; 3 Lemon Science and Technology Institute of Anyue County, Ziyang 642350, China

**Keywords:** lipoic acid, citrus, *Penicillium italicum*, natural compound, defense capability

## Abstract

Blue mold caused by *Penicillium italicum* (*P. italicum*) is a major postharvest disease in citrus fruits. Lipoic acid (LA) is a potent antioxidant with biological activity that was evaluated for its inhibitory effects on *P. italicum* and citrus blue mold using in vitro and in vivo experiments. The results demonstrated that LA effectively suppressed the mycelial growth and spore germination of *P. italicum*. LA increased hydrogen peroxide levels, compromising cell membrane integrity and leading to enhanced membrane permeability, as indicated by the increased relative conductivity and decreased protein and total sugar contents in *P. italicum* mycelia. Furthermore, LA delayed disease progression in citrus fruits infected with *P. italicum* through increasing total phenol and flavonoid contents and enhancing the activities of phenylalanine ammonia lyase, polyphenol oxidase, superoxide dismutase, and peroxidase in citrus peel. Overall, LA exhibited strong antifungal activity against *P. italicum* and improved citrus fruit resistance to blue mold, highlighting its potential as a natural postharvest disease control agent.

## 1. Introduction

Citrus fruits are highly susceptible to postharvest fungal infections, including *Penicillium digitatum*, *Penicillium italicum* (*P. italicum*), *Colletotrichum gloeosporioides*, and *Alternaria citri* [1,2,3]. Among these, blue mold induced by *P. italicum* and green mold caused by *P. digitatum* are the most destructive, causing severe economic losses [4,5]. Blue mold is of particular concern because it can also spread rapidly in packaged citrus stored at low temperatures, posing a significant risk worldwide [6]. Recent expansion in citrus production has resulted in seasonal surpluses and increased pressure on the fresh fruit market [7], necessitating effective preservation strategies. Traditional physical methods such as heat treatment, refrigeration, and ionizing radiation can provide limited control of postharvest diseases [4]. Although synthetic fungicides remain the most economical and widely used approach [8], the development of resistance in pathogens reduces their efficacy [9]. Additionally, synthetic fungicides pose risks to non-target microorganisms, food safety, human health, and the environment [10,11]. In response to the growing consumer demand for natural alternatives to maintain fruit quality and ensure food safety [12], developing safe and effective preservation methods to replace synthetic fungicides is crucial for the citrus industry.

Lipoic acid (LA) is a natural vitamin-like compound and a small-molecule coenzyme essential for aerobic metabolism in animals and is known for its antibacterial properties [13,14]. As a potent antioxidant, LA mitigates oxidative damage induced by heavy metals, chemical pollutants, and environmental toxins [15,16]. Initially isolated from pig liver in 1951 [17], Gorąca et al. found that LA was widely distributed in both animals and plants, with spinach being the richest plant source, as well as kidneys and livers having the highest concentrations in animals [18]. Recent studies have highlighted the antibacterial effects of LA. Levent et al. confirmed that α-LA exhibited antibacterial capacity against *Salmonella typhimurium* DT104 and *Escherichia coli* O157:H7 [19]. Chen et al. developed a tissue adhesive modified with LA and polyethylene glycol bisacrylate, which exhibited excellent biocompatibility, antimicrobial properties, and wound-healing potential [20]. Zhou et al. modified the natural antimicrobial peptide (LA-Bac8c) with LA as a hydrophobic ligand, further enhancing its antibacterial activity [21]. Shi et al. also reported that LA exhibited antimicrobial effects against *Cronobacter sakazakii* [22]. Despite these findings, the application of LA for postharvest citrus fruit preservation remains unexplored.

This study conducted both in vitro inhibition experiments and in vivo inoculation tests to evaluate the potential of LA to inhibit postharvest blue mold in citrus fruits and explore its underlying mechanism of action. The findings offer a theoretical foundation for the development of natural citrus preservatives as alternatives to chemical fungicides.

## 2. Materials and Methods

### 2.1. Materials

*P. italicum* was sourced from researchers at the South China Botanical Garden, Chinese Academy of Sciences. Lipoic acid (Compound: 6112) was acquired from Yihui Biotechnology Co., Ltd. (Shanghai, China). Fruits (*Citrus reticulata* Blanco cv. Ponkan) at the nine-tenths ripe stage were obtained from a well-managed orchard located at a latitude of 29.762964° N and a longitude of 106.367665° E in Beibei, Chongqing, China. Fruits free pests and mechanical damage were selected, cleaned with tap water, and allowed to air-dry at ambient temperature. Following in vivo inoculation, citrus peels were cryogenically frozen in liquid nitrogen and subsequently stored at −80 °C. Each experimental group included three biological replicates.

### 2.2. Measurement of Antifungal Activity and Spore Germination Rate

The in vitro antifungal effectiveness of LA was evaluated by the mycelial growth rate method [23]. Potato dextrose agar (PDA) medium was formulated with LA at concentrations of 0.4, 0.8, 1.6, and 3.2 mg mL^−1^, with the PBS buffer serving as the control (CK). A sterile punch (5 mm diameter) was employed to make a hole in the center of the medium, into which 50 μL of *P. italicum* spore suspension (1 × 10^6^ spores mL^−1^) was introduced. The medium was cultured at 26 °C for 7 days. The inhibition rate was calculated by subtracting the colony diameter of the treatment from that of the control, then expressed as a percentage of the control colony diameter. The virulence regression equation and median effective concentration (EC_50_) were established using the mycelial growth inhibition method outlined by Song et al. [24].

PDA disks (5 mm × 2 mm) were prepared by making holes in the center of PDA medium containing LA at concentrations of 0.4, 0.8, 1.6, and 3.2 mg mL^−1^ using a sterile punch (5 mm diameter). Subsequently, a 50 μL of *P. italicum* spore suspension (1 × 10^7^ spores mL^−1^) was applied to the PDA disks and cultured at 26 °C. Spore germination was observed under an optical microscope (Olympus Corporation, Tokyo, Japan) after 6, 12, 24, 36, and 48 h. A spore was regarded as germinating when its germ tube length exceeded its width. A minimum of 300 spores were counted in a randomized field of view. The spore germination rate was calculated as the percentage of the number of germinated spores to the total number of spores observed.

### 2.3. Measurement of Relative Conductivity, Total Sugar Content, Protein Content, and Hydrogen Peroxide (H_2_O_2_) Levels

Relative conductivity was detected following the methodology outlined by Kong et al. [25]. PDA disks (5 mm × 2 mm) were obtained from the margins of well-grown *P. italicum* colonies using a sterile punch. Fifteen PDA disks were placed in solutions containing LA at concentrations of 0.8, 1.6, and 3.2 mg mL^−1^. A DDS-307 conductivity meter coupled with a DJS-1C platinum-black electrode (Leichi Co., Ltd., Shanghai, China) was employed to measure the conductivity (C_0_). Measurements were taken at 1 h intervals during incubation. After boiling, the conductivity (C_1_) was measured again. Relative conductivity was calculated as C_0_/C_1_ × 100%.

The spores of *P. italicum* (1 × 10^6^ spores mL^−1^) were cultured in PDB (PDA without agar) medium containing LA at concentrations of 0.8, 1.6, and 3.2 mg mL^−1^ for 6, 9, 12, and 15 h. After centrifugation, the obtained *P. italicum* mycelia were repeatedly washed to discard the medium and then lyophilized to constant weight in a vacuum freeze-dryer (Songyuan Huaxing Technology Development Co., Ltd., Beijing, China). The total sugar content was determined using the anthrone colorimetric method outlined by Chang et al. [26]. Specifically, 0.1 g of mycelia was ground in an ice bath, mixed with 50 mL of water, and extracted by heating for 15 min. After cooling, 0.5 mL of 10% lead acetate solution was introduced, followed by adding 0.1 g of oxalic acid. The mixture was then filtered to obtain the supernatant for measurement. The protein content was detected by the color-deepening characteristics of protein combined with Coomassie Brilliant Blue dye [27]. Briefly, mycelia (0.1 g) was ground in an ice bath, diluted to 10 mL, and centrifuged to obtain the supernatant for analysis. Similarly, 0.1 g of mycelia was mixed with 4 mL of 50% trichloroacetic acid and centrifuged to extract the supernatant for measuring the H_2_O_2_ content [28]. The results were expressed as µg g^−1^ for total sugar, mg g^−1^ for protein, and mmol g^−1^ for H_2_O_2_.

### 2.4. P. italicum Infection of Citrus Fruits

Citrus fruits were cleaned with sterile water, dried, and then a wound with a diameter of 2 mm and a depth of 2 mm was artificially created [29]. Into the wound, 8 μL of *P. italicum* spore suspension (1 × 10^7^ spores mL^−1^) was inoculated. After air-drying, 8 μL of LA solution (1 mg mL^−1^, 2 × EC_50_) was injected into the hole, while CK received an equal volume of PBS buffer. The fruits were allowed to air-dry for 3 h and were then wrapped in film bags and placed in an incubator at 25 °C. Lesion diameters were measured and photographed on days 1, 2, 3, and 4. Three biological replicates, each containing thirty fruits, were analyzed.

### 2.5. Malondialdehyde (MDA) Content

Citrus peel powder (0.2 g), ground with liquid nitrogen, was mixed with 1.5 mL of 5% (*w*/*v*) trichloroacetic acid solution [30]. After centrifugation, the supernatant was collected and used for MDA detection. The unit was μmol g^−1^.

### 2.6. Total Phenol (TPC) and Total Flavonoid (TFC) Content

Approximately 1 g of citrus peel was resuspended in 80% ethanol and subjected to ultrasound extraction for 1 h. The resulting mixture was then centrifuged, and the supernatant was collected for determining the TPC using the Folin–Ciocalteu method [31] and TFC by the aluminum nitrate method [32]. The results were expressed as mg g^−1^.

### 2.7. Activities of Enzymes

Defense-related enzyme activity was assessed using assay kits obtained from Grace Biotechnology Co., Ltd. (Suzhou, China). Phenylalanine ammonia lyase (PAL) activity was assessed using L-phenylalanine as the substrate. The catechol method was employed to quantify polyphenol oxidase (PPO) activity. Superoxide dismutase (SOD) activity was quantified using the WST-8 method. Peroxidase (POD) activity was detected through the oxidation reaction of hydrogen peroxide and guaiacol. These units were U g^−1^.

### 2.8. Data Analysis

In this study, data were presented as the mean ± standard error from three replicate assays. Statistical analysis was conducted using a one-way analysis of variance (*p* < 0.05) and an independent samples *t*-test (*p* < 0.05 and *p* < 0.01) in SPSS 26, with figures created in Origin 2021.

## 3. Results and Discussion

### 3.1. In Vitro Experiments

#### 3.1.1. LA Inhibited the Growth of *P. italicum*

**Figure 1:**
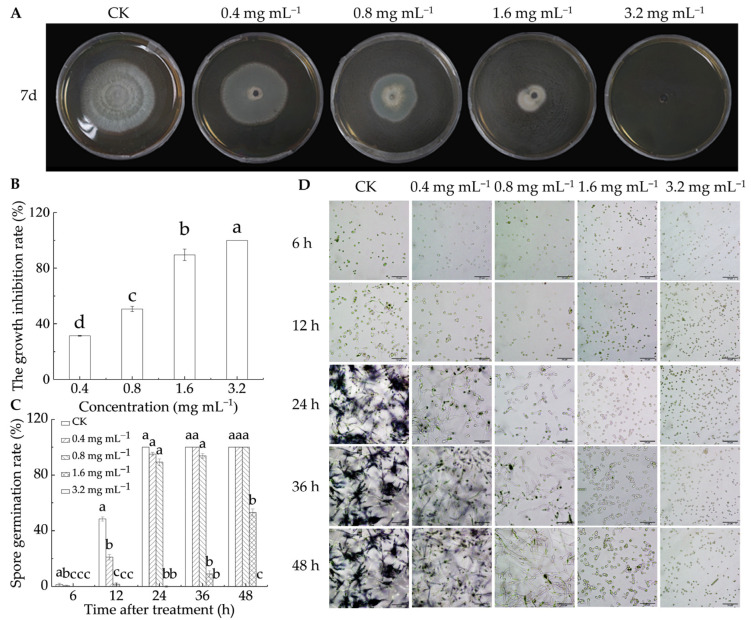
Antifungal capability of lipoic acid on *P. italicum* in vitro. (**A**) Inhibition effect; (**B**) mycelia growth inhibition rate; (**C**) spore germination rate; (**D**) spore germination micrograph (bar = 50 μm). Different letters denote significant differences among groups (*p* < 0.05).

As illustrated in Figure 1A,B, LA treatment effectively suppressed the growth of *P. italicum* after 7 d of in vitro culture, with antifungal activity increasing as the LA concentration increased. At 0.4 mg mL^−1^, the mycelial growth inhibition rate was 31.4%, rising to 50.6% at 0.8 mg mL^−1^, 89.6% at 1.6 mg mL^−1^, and reaching 100% at 3.2 mg mL^−1^. Figure 1C,D illustrate that LA effectively inhibited *P. italicum* spore germination. At 24 h, all spores in the control group had germinated, whereas no germination was witnessed in the 1.6 and 3.2 mg mL^−1^ groups. After 48 h, 53.1% of the spores in the 1.6 mg mL^−1^ treatment group germinated, while no germination occurred in the 3.2 mg mL^−1^ group. Additionally, a linear relationship was observed between the logarithm of the LA concentration and the probability of mycelial growth diameter, with the virulence regression equation fitting well (Table 1). The EC_50_ was 0.574 mg mL^−1^, and the smaller the value, the stronger the toxicity. The results showed that LA had strong antifungal activity, which aligned with the conclusions of Shi et al. [22] and Levent et al. [19].

#### 3.1.2. LA Damaged Cell Membrane Integrity of *P. italicum*

**Figure 2:**
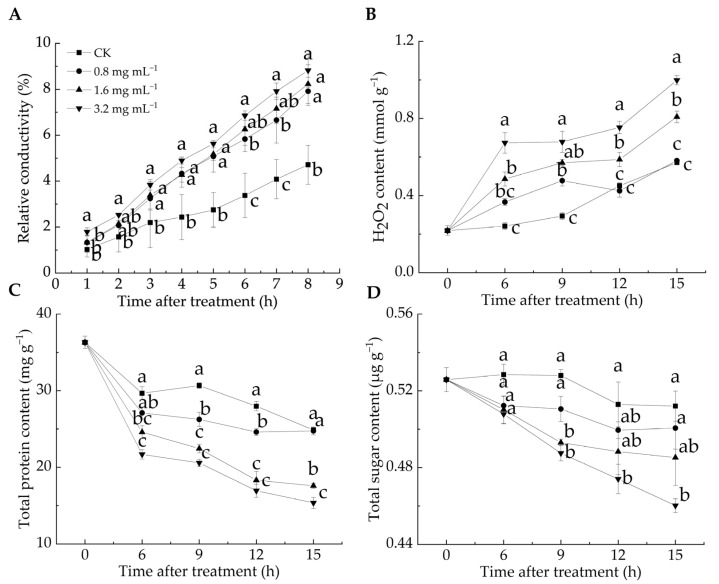
Effect of lipoic acid on relative conductivity (**A**), H_2_O_2_ level (**B**), total protein content (**C**), and total sugar content (**D**). Different letters denote significant differences among groups (*p* < 0.05).

Cell membrane permeability reflects membrane integrity and can be assessed by measuring relative conductivity [33]. H_2_O_2_ is a key reactive oxygen species (ROS), and excessive ROS can induce lipid peroxidation, compromising cell membrane integrity [12]. ROS levels serve as chemical indicators for evaluating the degree of cell membrane oxidation [34]. The relative conductivity of all LA-treated groups was significantly higher than that of the CK group from 3 to 8 h (*p* < 0.05) (Figure 2A). After 8 h, the relative conductivity in the CK group was 4.71%, whereas in the 0.8, 1.6, and 3.2 mg mL^−1^ groups, it reached 7.91%, 8.23%, and 8.82%, respectively, with the highest value observed in the 3.2 mg mL^−1^ group. These results suggested that the LA treatment elevated the relative conductivity of *P. italicum* mycelia, leading to electrolyte leakage and higher cell membrane permeability. Figure 2B illustrates that the H_2_O_2_ content in the 1.6 and 3.2 mg mL^−1^ groups was significantly higher than that in the CK group during the entire incubation period, whereas no significant difference was found between the 0.8 mg mL^−1^ and CK groups at 6, 12, and 15 h (*p* < 0.05). These findings suggested that the LA treatment induced substantial H_2_O_2_ production, disrupting the cell membrane integrity of *P. italicum* mycelia, as evidenced by the increased relative conductivity.

Proteins and sugars are essential for maintaining normal physiological metabolism in mycelia, and their loss reflects increased cell membrane permeability [35]. As shown in Figure 2C,D, all groups exhibited a general decline in protein and total sugar contents over the incubation period, with the CK group consistently maintaining higher levels than the LA-treated groups. During incubation, the protein content decreased by 31.5% in the CK group, 31.8% in the 0.8 mg mL^−1^ group, 51.6% in the 1.6 mg mL^−1^ group, and 57.7% in the 3.2 mg mL^−1^ group, respectively. Notably, the protein content in the 1.6 and 3.2 mg mL^−1^ groups was significantly lower than that in the CK group throughout the incubation period (*p* < 0.05). Similarly, the sugar content decreased by 2.6% in the CK group, 4.8% in the 0.8 mg mL^−1^ group, 7.7% in the 1.6 mg mL^−1^ group, and 12.5% in the 3.2 mg mL^−1^ group, with a significant reduction in the 3.2 mg mL^−1^ group compared with the CK group, except at 6 h after the treatment (*p* < 0.05). Overall, the LA treatment led to a decrease in the protein and total sugar content of *P. italicum* mycelia, indicating cell membrane damage. This result aligned with the findings of Song et al. [24], who found that disruption of the mycelial cell membrane structure caused intracellular substance leakage and increased extracellular sugar and protein contents.

The destruction of cell membrane integrity can generate physiological disorders and even cell death of fungi, making this a key mechanism for various natural substances to exert antifungal activity [12,36]. Prolonged stress can compromise an organism’s defense mechanisms, leading to a sharp increase in ROS, which disrupts normal cellular functions. Excessive ROS accumulation oxidizes biomacromolecules, ultimately resulting in cell membrane damage or cell death [37]. Shi et al. demonstrated that LA exerted antibacterial effects by inducing cell membrane dysfunction and morphological alterations in *Cronobacter sakazakii* [22]. Yang et al. discovered that LA depolarized the cell membrane potential of *Yersinia enterocolitica*, significantly reducing the intracellular pH and ATP levels, which disrupted the membrane structure [38]. Figueroa et al. reported that LA induced cytoplasmic protein loss in *Thecaphora frezzii*, leading to reduced ergosterol levels and alterations in the cell wall and membrane of fungi, ultimately causing cell death [39]. Therefore, LA inhibited the growth of *P. italicum* mycelia by compromising cell membrane integrity.

### 3.2. In Vivo Experiments

#### 3.2.1. LA Treatment Delayed the Decay Symptoms of Citrus Fruits

**Figure 3:**
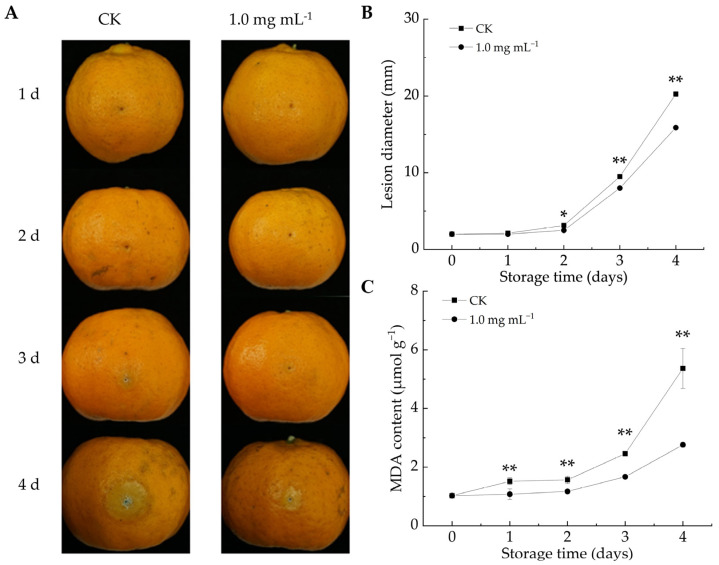
Effect of lipoic acid on postharvest disease spot extension in Ponkan fruit. (**A**) Disease progression; (**B**) lesion diameter of *P. italicum*-infected fruits; (**C**) MDA content. * denotes *p* < 0.05; ** denotes *p* < 0.01.

As shown in Figure 3A, the decay area of citrus fruits increased in both groups throughout the storage duration. After 2 d of infestation, the lesion diameter in the LA-treated group was notably reduced compared to the CK group (*p* < 0.05). By the fourth day, the lesion diameter in the CK group reached 20.46 mm, whereas the fruits in the LA-treated group had a lesion diameter of 15.94 mm, representing a 22.1% reduction. Overall, LA treatment effectively reduced lesion size and mitigated decay symptoms in *P. italicum*-infected fruits. Similarly, berberine [12] and ε-poly-l-lysine [40] could inhibit lesion expansion in citrus fruits infected with *P. italicum*. These findings highlight the potential of natural compound-based preservation strategies for enhancing disease resistance and extending postharvest fruit storage.

#### 3.2.2. LA Reduced MDA Content in Citrus Peel

MDA can serve as an indicator of membrane damage severity and influence membrane permeability [41]. MDA content is negatively correlated with fruit disease resistance [12]. As the storage duration increased, MDA levels increased in both the CK and LA-treated groups (Figure 3C). However, LA treatment significantly reduced MDA accumulation, with the average MDA content from days 1 to 4 being 33.7% lower than that in the CK group (*p* < 0.01). Biewenga et al. observed that LA possessed ROS-scavenging properties, promoted endogenous antioxidant regeneration, facilitated oxidative damage repair, and mitigated stress-induced damage [42]. These results indicated that LA treatment decreased the MDA content in *P. italicum*-infested citrus fruits, alleviated oxidative damage, and enhanced fruit resistance. Similarly, Song et al. reported that the delayed progression of fungal infections in harvested fruits was associated with suppressed oxidative damage [24].

#### 3.2.3. LA Increased Defense-Related Enzyme Activities in Citrus Peel

**Figure 4:**
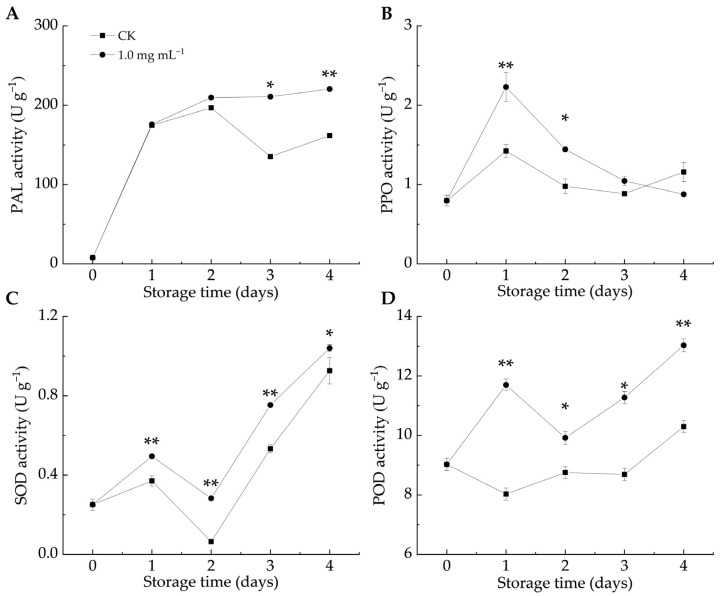
Effect of lipoic acid on activities of PAL (**A**), PPO (**B**), SOD (**C**), and POD (**D**). * denotes *p* < 0.05; ** denotes *p* < 0.01.

Defense enzymes like PAL, PPO, SOD, and POD are crucial in plant–pathogen interactions. PAL contributes to stress responses by affecting the biosynthesis of flavonoids, lignin, and phytoalexins [43]. When fruits and vegetables are infected by pathogens, PPO activity increases, leading to the production of lignin and phenols that help resist pathogen invasion [12]. In both groups, the PAL, SOD, and POD activities increased as the infestation progressed, whereas the PPO activity initially increased before gradually declining. As shown in Figure 4A, the PAL activity in the LA-treated group remained higher than that in the CK group, with significant differences observed on days 3 and 4 (*p* < 0.05). Similarly, the PPO activity in the LA-treated group was higher than that in the CK group (Figure 4B), with significant differences between days 1 and 2 (*p* < 0.05). These findings indicated that LA treatment enhanced the PAL and PPO activities in citrus peels, promoting the accumulation of defense-related metabolites and triggering the fruit defense response.

ROS accumulation serves as both a defense barrier against pathogen invasion and a signaling mechanism to activate plant defense responses [44]. The total phenol and protein contents, along with antioxidant enzyme activities, can help counteract the toxic effects of ROS in host plants [45]. In the enzyme-mediated antioxidant system, SOD catalyzes the conversion of O_2_^–•^ to H_2_O_2_, which is then transformed into oxygen and water by the action of POD and CAT [46]. POD also contributes to plant defense by catalyzing the final step of lignin biosynthesis [44] and inhibits pathogen growth by oxidizing phenols into harmful quinones [47]. Figure 4C,D illustrate that LA significantly promoted SOD and POD activities compared to the CK group throughout storage (*p* < 0.05). Similarly, *Pichia caribbica* scavenges ROS through enzymatic and non-enzymatic mechanisms, enhancing disease resistance in cherry tomatoes [48]. These findings suggested that LA treatment enhanced SOD and POD activities, mitigated oxidative damage, and improved the resistance of citrus fruits to blue mold.

#### 3.2.4. LA Increased TFC and TPC Contents in Citrus Peel

**Figure 5:**
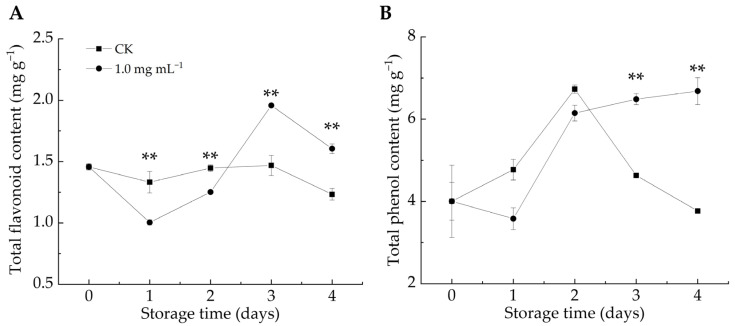
Effect of lipoic acid on total flavonoid (**A**) and total phenol content (**B**). ** denotes *p* < 0.01.

The phenylpropanoid metabolic pathway plays a crucial role in plant defense by generating bioactive compounds (e.g., phenols, flavonoids, salicylic acid, and lignin), which contribute to fortifying host cell structures against fungal infections [49,50]. Additionally, phenol production induces the synthesis of pathogenesis-related proteins (e.g., chitinase, 1,3-glucanase, and thaumatin), which are positively correlated with plant resistance [45]. As shown in Figure 5A,B, the TFC and TPC contents in the CK group exhibited an overall decline, whereas the LA-treated group exhibited an increasing trend. After 3–4 d of infestation, LA significantly stimulated TPC accumulation compared with the CK group (*p* < 0.01). After 1–2 days of infestation, the TFC content in the LA-treated group was considerably lower than that in the CK group, but it became significantly higher after 3–4 days (*p* < 0.01). We postulated that in the early stage, LA treatment effectively mitigated decay symptoms in *P. italicum*-infected fruits and protected them from experiencing pathogen-induced stress, thus delaying the activation of defense responses in citrus fruits, resulting in higher TFC content in the CK group than in the LA-treated group. Overall, LA treatment enhanced the TFC and TPC contents in citrus peel, strengthening the resistance against *P. italicum.* Numerous investigations have demonstrated the antimicrobial properties of phenolic compounds and flavonoids [24].

## 4. Conclusions

LA effectively suppressed the mycelial growth and spore germination of *P. italicum*, and its inhibitory effect increased in a dose-dependent manner. The increase in H_2_O_2_ content and relative conductivity coupled with the reduction in protein and total sugar levels in *P. italicum* mycelia after LA treatment suggested that LA disrupted fungal growth by inducing ROS accumulation and compromising cell membrane integrity. Furthermore, LA delayed disease progression in *P. italicum*-infected citrus fruits, likely by enhancing defense-related enzyme activity and promoting the accumulation of defense-related metabolites in citrus peel, which activated the fruit defense response. Overall, LA displayed potent antifungal activity against *P. italicum* and enhanced citrus fruit resistance to infection. As a natural compound, LA is a promising approach for preventing and controlling postharvest citrus diseases, thereby contributing to the healthy development of the citrus industry.

## Figures and Tables

**Table 1:** Toxicity of lipoic acid against *P. italicum*.

Virulence Regression Equation/(y)	Phase Relation (r)	Median Effect Concentration (EC_50_)	95% Confidence Interval
y = −0.357 − 1.483x	0.858	0.574 mg mL^−1^	0.448–0.738

## Data Availability

The original contributions presented in this study are included in the article. Further inquiries can be directed to the corresponding author.
